# The circuits of healthcare: Understanding healthcare seeking behaviour—A qualitative study with tuberculosis patients in Lisbon, Portugal

**DOI:** 10.1371/journal.pone.0261688

**Published:** 2021-12-28

**Authors:** Rafaela M. Ribeiro, Philip J. Havik, Isabel Craveiro

**Affiliations:** Global Health and Tropical Medicine, GHTM, Instituto de Higiene e Medicina Tropical, IHMT, Universidade Nova de Lisboa, UNL, Lisboa, Portugal; The University of Georgia, UNITED STATES

## Abstract

**Background:**

Understanding health delivery service from a patient´s perspective, including factors influencing healthcare seeking behaviour, is crucial when treating diseases, particularly infectious ones, like tuberculosis. This study aims to trace and contextualise the trajectories patients pursued towards diagnosis and treatment, while discussing key factors associated with treatment delays. Tuberculosis patients’ pathways may serve as indicator of the difficulties the more vulnerable sections of society experience in obtaining adequate care.

**Methods:**

We conducted 27 semi-structured interviews with tuberculosis patients attending a treatment centre in a suburban area of Lisbon. We invited nationals and migrant patients in active treatment to participate by sharing their illness experiences since the onset of symptoms until the present. The Health Belief Model was used as a reference framework to consolidate the qualitative findings.

**Results:**

By inductive analysis of all interviews, we categorised participants’ healthcare seeking behaviour into 4 main types, related to the time participants took to actively search for healthcare (patient delay) and time the health system spent to diagnose and initiate treatment (health system delay). Each type of healthcare seeking behaviour identified (inhibited, timely, prolonged, and absent) expressed a mindset influencing the way participants sought healthcare. The emergency room was the main entry point where diagnostic care cascade was initiated. Primary Health Care was underused by participants.

**Conclusions:**

The findings support that healthcare seeking behaviour is not homogeneous and influences diagnostic delays. If diagnostic delays are to be reduced, the identification of behavioural patterns should be considered when designing measures to improve health services’ delivery. Healthcare professionals should be sensitised and perform continuous capacity development training to deal with patients´ needs. Inhibited and prolonged healthcare seeking behaviour contributes significantly to diagnostic delays. These behaviours should be detected and reverted. Timely responses, from patients and the healthcare system, should be promoted.

## Background

Unfortunately, Tuberculosis (TB) is not a disease of the past, in 2019 it killed 1.4 million people worldwide [1]. The WHO European region accounts for 2.5% of global disease burden [1]. In the European Union, Portugal boasts one of the highest TB incidence, despite its decreasing trend [2]. With a best estimate of 24 incident cases per 100.000 inhabitants, the country totalled 2137 notified cases in 2018 [2]. For the same year, 57.3% of TB cases were concentrated in the biggest urban settings of Lisbon and Oporto [3]. The association between TB and socio-economic factors determining higher TB rates affecting impoverished urban settings is well-known [4, 5].

Pulmonary TB is an infectious air-born disease and the most common type of TB [6]; around 16% of cases may affect other organs [1]. It may present a subtle onset and a non-specific array of symptoms [6], which contribute to potential delays in its detection. TB *total delay* is divided in *patient delay*–time a person takes to seek a first contact with healthcare, and *health system delay*–time passed from the first contact with healthcare until appropriate treatment initiation [7]. Measuring delays until treatment initiation assists in evaluating TB management at service delivery level. In Portugal, a study including 6838 TB patients (2010–2014), detected an average patient delay of 33 days, 17 for health system delay and 68 for total delay [8]. Other national studies, showed that big cities, such as Lisbon, present significant geographical variations in TB delays [5, 9]. TB tends to cluster in “hotspots”.

The End TB strategy establishes milestones for TB control until 2030 [10]. Its first pillar embraces patient-centred care—a step towards a people-centred model of healthcare delivery, where social, economic and, cultural aspects affecting illness are highlighted [11]. A patient-centred approach implies addressing people´s lifestyles, and furthermore, raises the possibility of dealing with unmet non-medical needs (as proper housing or employment) influencing health status [12]. The study of patients´ pathways to care encompasses underlying issues affecting patients´ experiences and perceptions related to illness [13–15].

The initial action towards seeking medical help, *Healthcare Seeking Behaviour* (HCSB), is a key component of a patients’ pathway, and is embedded in the “complex system” of healthcare delivery [16]. HCSB defined as a “tool for investigating the individual´s or population´s interaction with the health system” is fit-for-purpose to study how people perceive health, and how they access and use the available services to promote it [17]. Challenges associated with implementing interventions may derive from neglecting specific contextual mechanisms influencing people´s behaviour [18, 19].

While the analysis of HCSB provides information about the interaction between patients and the health system, the analysis of individual dimensions of illness can be framed by the *Health Belief Model* (HBM) [13]. It is a widely used social cognitive model, originally designed in the context of a failed TB screening program in the 1950´s [20]. The HBM enables exploring a person´s agency based on their illness perceptions, in its components of perceived susceptibility, -severity, -benefits, -barriers, cues to action and self-efficacy [21].

In Portugal, qualitative published research has integrated physicians’ perspectives on TB patients´ management [22]. Nevertheless, the TB patient´s perspective on their own pathways to care, including nationals and migrants remains, as yet, unexplored. These trajectories assist in illustrating the difficulties the poorer sections of society experience in obtaining adequate care [23].

We first proceed to analyse the circumstances, experiences, perceptions, and behaviours of TB patients in their initial search for healthcare. Secondly, we ascertain the main entry points, routes of care and bottlenecks inducing delays in TB treatment initiation. By exposing “critical points” of these circuits and considering possible reasons for them, we wish to highlight the need to embrace patients´ perspectives when implementing quality improvements of health services and interventions [24].

## Methods

### Study design

This is a qualitative study, embedded on a project based upon a case study on a treatment Centre for Lung Diseases (*Centro de Diagnóstico Pneumológico*—CDP) in Lisbon. The CDP is a medical unit, focused on out-patient TB management The project is a mixed-method single case study [25]. Here we focus on a share of the qualitative information retrieved in relation to healthcare seeking behaviours of participants.

### Study setting

The CDP object of study is located in the north of the Greater Metropolitan Area of Lisbon. Its coverage area comprised three municipalities with an approximate population of 600.000 inhabitants in 2016 [26, 27]. One of these municipalities (Amadora) boasts the highest rate of risk factors for TB in Portugal [28]. An unpublished internal CDP´s report, referred a TB incidence of 28.6 cases per 100.000 inhabitants for the whole area in 2016, whilst recording 39.3 for Amadora. The same report lists a total of 185 notified cases, 41.0% of which foreign-born.

The Portuguese National Health Service (SNS) provides universal access for Portuguese citizens and legal migrants [29]. Primary Healthcare (PHC) functions as the system´s first tier encompassing an assigned MD who also acts as a gatekeeper for specialised care [29]. In recent years, the debate on equal access to PHC has signalled the large number of SNS users without an assigned MD [30]. Although this problem has been tackled by authorities resulting in 93.0% of registered citizens having an assigned MD in 2018, in Amadora 26.1% of registered citizens lacked one [31]. Nevertheless, urgent consultations with a general MD are possible at PHC level, and hospital emergency room (ER) assistance can be accessed upon users’ personal initiative.

Migrants whose legal status is irregular, and are not registered in the SNS, face greater barriers to public healthcare access [32]. Nevertheless, when vital, urgent or of public health concern health problems, such as TB, are present, access is permitted and it is free of charge [33]. For situations not included in exemptions, out-of-pocket fees are charged [34]. TB notification is mandatory and monitored by a surveillance system—SVIG-TB which gathers information on patients´ demographic characteristics, risk groups, comorbidities, clinical, radiological and microbiological characteristics of TB, and *TB delays* [35].

### Data collection

Semi-structured interviews were conducted with TB patients. A flexible topic guide was pilot tested providing insights on interview´s duration, probes and silences, and hunches on how to modify questions to get richer answers. RR conducted all interviews; the experience of being a family doctor and a PhD student provided the necessary training. Moreover, RR has worked in healthcare centres with persons without an assigned MD, in the Lisbon area, and with TB patients in Mozambique. The interview guide is annexed as “S1 Appendix”.

Inclusion criteria were, age 18 years or over, receiving active TB treatment and attending the CDP between October 2018 and January 2019. Exclusion criteria were severe illness at presentation. The CDP pneumologist´s opinion was considered when inviting patients. Two different sampling techniques were used to gather more information about recent migrants: a) purposive sampling: targeting migrant patients arriving in Portugal during the previous 2 years (2017 and 2018); b) convenience sampling: encompassing all patients meeting the inclusion criteria and scheduled for a consultation. Interviews were held in a private room, in a one-time, one-to-one format, lasting about 40 minutes, following TB consultations. We audio-recorded interviews, and if permission was withheld interview notes were taken. Twenty-seven patients were interviewed during 3 months field work, after which data collection appeared to have reached saturation, as people´s narrative did not seem to bring new information on key topics [36].

### Data analysis

Recorded interviews were transcribed *verbatim*. RR used manual descriptive thematic analysis to assess the transcripts [37]. The initial coding frame was developed independently by IC and RR based on the first 6 interviews (22%); differences were resolved by discussion and thematical consensus generation was achieved. Further coding was done by RR. An Excel spreadsheet (*Microsoft*, *Redmond*, *WA*, *USA)* was used as a software tool to assist codification and analysis.

Data was analysed inductively and deductively following a constructivist approach as we focused on participants´ personal experiences and perceptions for generating data. Comparisons were made between interviews to establish common patterns and differences in the data [38]. The inductive information was saturated in the course of a theory building process: enough examples were provided to exemplify each conceptual category [36]. Clinical processes were checked to match time lapses between participants´ discourse and clinical registers. Quotes were translated and grammatically corrected. The application of HBM emerged during the analysis stage, being used as a conceptual explanatory framework to consolidate the findings [21]. The research team comprised a female, family doctor and PhD student (RR), a female PhD sociologist and assistant professor (IC), and a male PhD anthropologist and assistant professor (PH). We used the consolidated criteria for reporting qualitative research (COREQ) attached as “S2 Appendix” [39].

### Ethical considerations

Participants were encouraged to talk freely; interruptions were avoided, since TB may touch upon sensitive personal issues. Written informed consent was obtained after the study´s goals and privacy statements, were explained. All data were anonymized. Files and informed consent are stored safely and confidentially. All raw data from the interviews will be destroyed when the project is concluded. Participants did not receive financial compensation for participating nor had access to the transcripts or feedback from the results. The project´s protocol was approved by the Ethics Committee of Lisbon and Tagus Valley Regional Health Administration (ARS-LVT—ref.11703/CES/2018).

## Results

Twenty-seven patients were included in the present study. Participants originated from Portugal, Angola, Guinea-Bissau, Cabo Verde, Brazil, and Romania. The average age was 38 and 33 the most frequent. HIV (Human Immunodeficiency Virus) was the main risk factor after being a migrant; other risk factors were alcohol misuse, smoking and diabetes. Most participants did not have an assigned MD in PHC. Purposive sample´s participants came from two countries, tended to be younger, female, have higher school level, more cases of extrapulmonary TB, and HIV infection (Table 1).

**Table 1:** Characteristics of study participants according to sampling technique.

	Purposive sample (Arrival in Portugal in 2018/2017)	Convenience sample
**Number persons listed for invitation** *	20	The ones showing up to a previously scheduled consultation
**Total number of participants**	12	15
**Nationalities**	Angola: 8	Portugal: 11
	Guinea-Bissau: 4	Brasil: 1
		Cabo Verde: 1
		Romania: 1
		Angola: 1
**Type of TB**	Pulmonary: 3	Pulmonary: 10
	Extrapulmonary: 9	Extrapulmonary: 5
**Gender (Female/Male)**	8/4	6/9
**Ages**	[19–36]	[20–86]
**Occupation**	4 business, 3 cook, one master student, nurse, grape harvest, unemployed and one refugee	4 mason, 2 university student, one healthcare assistant, cook, housewife, seamstress, electrician-retired, security guard, fitness instructor, school auxiliary and one unemployed
**University degree (complete or incomplete)**	8	2
**Number persons having an assigned family MD**	2	6
**Risk factors**	Migrant (all), 4 HIV, one health professional, unemployment, food insecurity	5 smoking, 3 alcohol misuse, 4 foreign born, 2 old age, 2 chronic lymphatic leucaemia, one HIV, one Portuguese migrant who lived in a high incidence country, TB childhood, healthcare professional, unemployment, depression, contact with mother who had TB, diabetes
**Participants**	P2, P3, P4, P7, P9, P15, P19, P20, P21, P23, P24, P29	P5, P6, P8, P10, P11, P12, P13, P14, P16, P17, P22, P25, P26, P27, P28

* From the purposive sample we excluded the pilot interview, as well as one person who refused to sign the informed consent after being interviewed, four persons refused to participate stating lack of time, and one person did not show up, one person was not invited because of bad health status. Three participants did not consent to audio recording. Note: foreign born participants in the convenience sample have been living in Portugal for more than 2 years.

**Fig 1:**
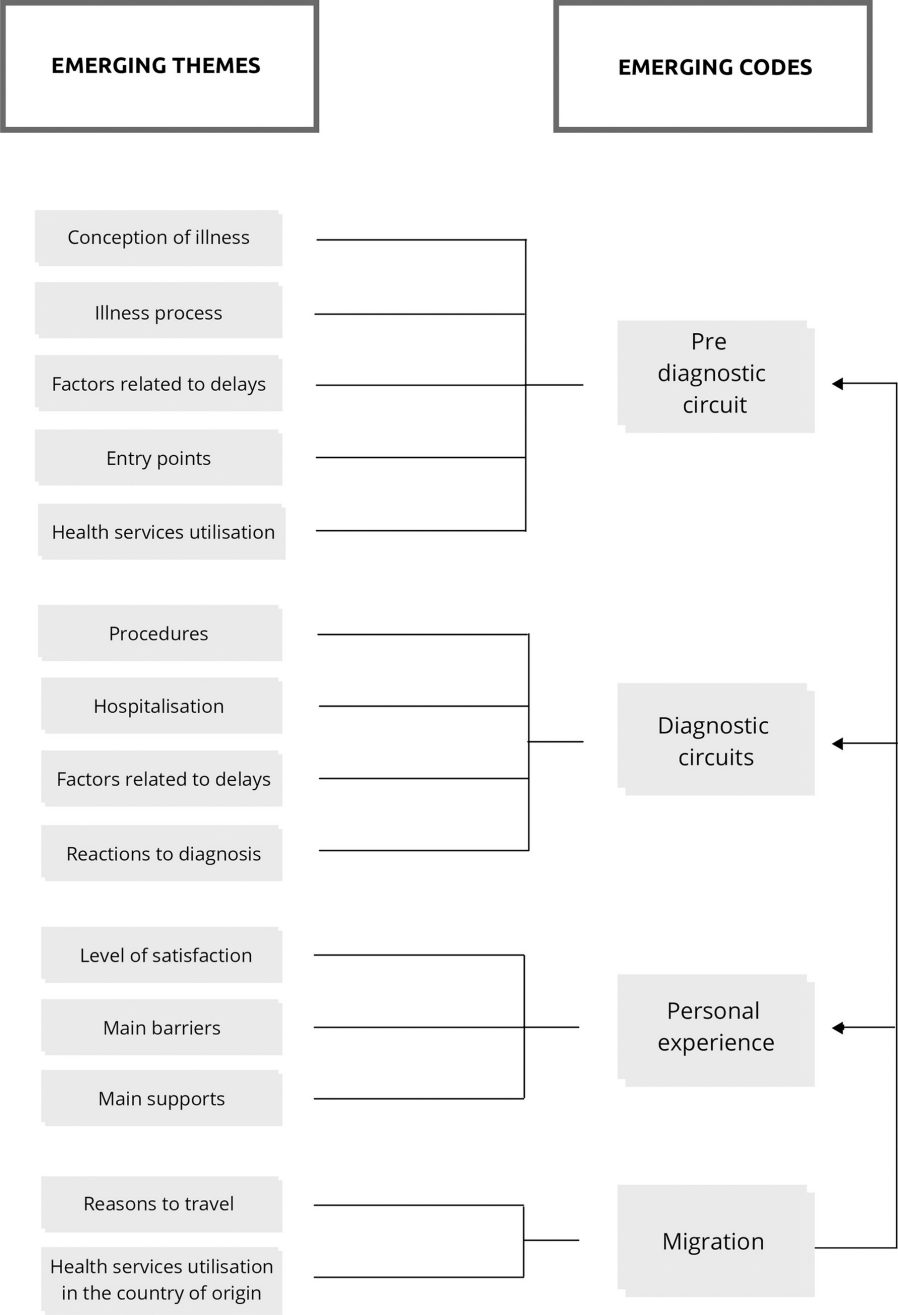
Main codes and themes emerging from the data deductively.

Thematic analysis was done deductively and inductively. Themes emerging deductively described patients´ circuits inside the healthcare system from initial symptoms until the interview moment (Fig 1). Inductively, we propose an analytical framework categorising HCSB, classified considering TB delays and, based upon participants’ accounts of seeking healthcare for this illness episode. This paper focuses on the inductive data.

Inductively, focusing on the interaction between patients and the healthcare system, we propose classifying HCSB into four categories: 1) inhibited HCSB, 2) timely HCSB, 3) prolonged HCSB and 4) absent HCSB. To classify HCSB we arbitrarily designated a patient delay of less than one month, and a health system delay of less than two months, as acceptable. To our knowledge there is no standard acceptable TB delay. For some participants coming from abroad, initial symptoms and the first consultation occurred elsewhere; we chose to account this time for delays in a patient-centred fashion. All justifying quotes are compiled as a supplementary document “S3 Appendix”.

### Inhibited HCSB–*major patient´s delay*

The five participants showing an *inhibited HCSB* tended to significantly delay seeking medical assistance. They took between two to six months feeling symptoms before deciding to seek medical help. Explanations offered included fear of hospitals and possible implications of diagnosis, the need to work to cover financial expenses, alcohol masking symptoms, assuming an attitude of inertia surrendered by the illness situation or motivated by HIV stigma.

*" Life is not that easy for us to be missing working days… I have bills to pay*, *I never went to the hospital… by that time*, *I also drank*, *I drank a lot*, *maybe the alcohol itself masked the deficiency the body had*.*” (P26*, *male*, *age 45*, *Portugal*)

All participants in this category chose the ER as an entry point, except one who had been previously diagnosed in other country, and opted for entry via the PHC, she was the only participant not being hospitalised. For all of them, health system delay was less than 2 months; most of them suffered from a pulmonary form of TB (Table 2).

**Table 2:** Participants´ delays, diagnostic circuits, and TB types, grouped according to their HCSB.

		*Patient delay*	*Health system delay* ^a^	*Diagnostic circuit in Portugal*	*Type of TB*	*Risk factors* ^b^	*Sam-ple*
**Inhibited HCSB**	P3	2–3 months	< 2 months	PHC—CDP	Dis	HIV	Pur
	P5	2–3 months	1 day	ER—H	P	Alcohol misuse, smoker	Con
	P8	6 months	1 ½ months	ER—H	Pleural	Smoker	Con
	P9	2 months	< 1 month	ER—H	P	-	Pur
	P26	3–4 months	< 1 month	ER—H	P	Alcohol misuse, smoker	Con
**Timely HCSB**	P6	Hours	1 week	ER—H	P	Smoker	Con
	P7	2–3 weeks	1 month	ER—H	Pleural	Food insecurity	Pur
	P11	2 weeks	< 1 month	PSC—H	P	HIV, Alcohol misuse	Con
	P13	1 month	< 1 month	PHC—CDP	P	Old age, migrant in Mozambique	Con
	P21	2 weeks	< 1 month	PHC—CDP	P	HIV	Pur
	P22	2–3 weeks	< 1 month	ER—H	Pleural	Contact with TB	Con
	P23	2 weeks	2 months	Private C—ER—H	P	Health professional	Pur
	P24	<1 month	2 months	PHC—ER—CDP	Pleural	Unemployed	Pur
	P27	<1 month	2 months	Private C—ER—H	Bone	-	Con
**Prolonged HCSB**	P4	1 month	> 8 months	**»** Private C–CDP–ER—H	Bone	Newly diagnosed HIV	Pur
	P10	<1 month	9 months	PHC **»** PSC—H	P	Old age, chronic lymph leuk	Con
	P14	1 year	> 1 year	PSC **»** ER—H	Lymphatic	Chronic lymph leuk	Con
	P15	1 month	4–5 months	**»** PHC **»** ER—H	Peritoneal	HIV	Pur
	P20	15 days	11 months	**»** ER—H	Bone	-	Pur
	P25	1 month	8 months	PHC—Private C **»** ER—H	Meningitis	-	Con
	P29	Months	> 1 year	**»** Private C **»** Private ER–Private H—CDP	Pleural	-	Pur
**Absent HCSB**	P2	One single consulta-tion	< 1 month	Active screening refugees–ER—H	Pleural	Refugee	Pur
	P12	Summon-ed by the CDP	< 1 week	Active screening of contacts (CDP)	P	Contact with infectious TB	Con
	P16	Demand for chest x-ray	7 months	Occupational health—PSC—CDP	P	Smoker, healthcare professional	Con
	P17	Only pharmacy	< 1 week	Charity Org—PHC—ER—H	P	Unemployment, depression	Con
	P19	No HCSB	< 1 month	Ambulance—ER—H	Dis	-	Pur
	P28	Only pharmacy	< 1 week	PSC—PHC—ER—H	P	Diabetes	Con

PHC = consultation in primary healthcare; CDP = centre for lung diseases; ER = emergency room; H = hospitalisation; PSC = specialist consultation in public service; Private C = consultation in the private sector; Private ER = emergency room in the private sector; Private H = hospitalisation in the private sector; Charity Org. = charitable organisation. P = pulmonary TB; Dis = disseminated TB; Chronic lymph leuk = chronic lymphatic leukaemia. Pur = purposive sample; Con = convenience sample.

^**a**^ healthcare system delay shown, for purposive sample, starts since the first consultation in home countries referred by participants (this part of the circuit is not shown).

^**b**^ being a migrant was not included as a risk factor, as this characteristic was highlighted with the sampling method. Although chronic lymphatic leukaemia is not a known risk factor for TB it causes immunosuppression which is a known risk factor for TB.

“**»**” symbol means a prolonged period has been spent in the diagnostic circuit due to health system delay, whether before arriving to Portugal, whether between medical consultations.

For these participants illness symptoms, such as cough or weakness, were unspecific and insufficient reason to trigger HCSB. The triggers for their HCSB were personal, such as fear of a sudden extreme physical pain, or externally induced as a “societal push”. Using the constructs of the health belief model to consolidate this behavioural typology, we infer that, perceived benefits of seeking healthcare were low, as well as perceived severity of disease, only until a strong event (cue to action) induced them to act (Table 3).

*“I thought it was only a flu, I ignored everything, I had a cough, 2 months, I was already feeling ashamed in the bus.” (P3, female, age 36, Guinea-Bissau)*

**Table 3:** Interactions between the Health Belief Model and four types of health seeking behaviour*. The individual perception of illness is influenced by participants´ contexts, and past and present health system´s response.

HBM Type HCSB	Perceived susceptibility	Perceived severity	Perceived benefits	Perceived barriers	Cues to action
**Inhibited HCS**	Neglection of symptoms	Perceived severity increases with time because of evident weight loss, compulsive cough, awareness of anorexia, extreme shortness of breath, extreme pain	No need to seek healthcare for “normal” symptoms; no benefits if it is necessary to lose a working day; maybe traumatic experience with healthcare	High perceived barriers to healthcare usage	Internal: fear of acute extreme disabling symptom
				Emergency room is the preferred place to go when extreme situation is present	External: social stigma (e.g. a cough making people stare in public), or community alert (e.g. a neighbour realises illness and speaks out)
**Timely HCSB**	High awareness and concern about symptoms	Severity sufficient to trigger action in relatively little time	There is a “trustful” relationship with healthcare, in a formal or informal way	Low perceived barriers as there seems to be a “familiarity” with the healthcare system	Internal: concerns about the initial symptoms of disease.
					External: accessibility to trusted healthcare.
**Prolonged HCSB**	High awareness and concern about symptoms	High perceived severity as symptoms worsens with time	High perceived benefits in receiving healthcare which motivates its pursuance in time	High perceived barriers, as healthcare is not able to fix suffering. No treatment is effective, neither a diagnosis is achieved	Internal: suffering caused by symptoms for a prolonged time and hope for a cure
					External: financial possibility of pursuing healthcare (namely migrants), a pre-scheduled consultation.
**Absent HCSB**	Varies. High if there is a search for a preventive service, or low if there is a tendency for inhibited HCSB	Although symptoms are absent or “neglected”, perceived severity is increased after “awareness”	There is a passive surrendering to healthcare	Low barriers to accessing healthcare as the health system is the one acting upon them	External: “push” from the health system as an “obligation”, a “protection”, a “responsibility” or an “advice”

* For detailed information about the inductive information retrieved see the supplementary material “S3 Appendix” with the exhaustive compilation of quotes.

### Timely HCSB–*appropriate action-response*

The nine participants acting early when symptoms appeared were classified as having a *timely HCSB*. Patient delay was less than one month. Although symptoms were unspecific, respondents mentioned early acute illness sensations inducing their action. Timely behaviour of participants related to an awareness of symptoms and, accessibility, or trust in different entry points of the healthcare system. A specialist consultation, a family MD well-known to the participant, or seeing a friend working as MD in a hospital, were all preferred entry points.

*" I have been around a week feeling this way then I went to the hospital… normally I go to the hospital where I received follow-up treatment for HIV because they have my clinical history.” (P11, male, age 39, Portugal)*

The ER and the private healthcare service were included in participants’ choices. For some migrant participants, coming to Portugal was motivated by the search for better healthcare (than in their home countries) and they acted quickly. Others, previously lived in Portugal or came as tourists, but upon falling ill, they sought healthcare.

*"I arrived [in Portugal] and I went directly to a consultation [in a private hospital]. I already knew it. I have always done my consultations there.” (P23, female, age 33, Angola)*

The diagnostic care cascade was initiated in a timely manner with a health system delay of less than 2 months. Three participants entering through PHC level were not hospitalised. Health system´s delay tended to be longer for participants opting for private care, and for another who was not hospitalised after the ER episode. The type of TB presented was mainly pulmonary or pleural (Table 2).

Through the HBM lens, this category of HCSB bears a relationship with healthcare systems in terms of proximity. Low perceived barriers to seeking healthcare combined with a high perceived severity of disease results in a timely HCSB (Table 3).

### Prolonged HCSB—*major healthcare system delay*

Seven participants showing a *prolonged HCSB* suffered persistence and worsening of symptoms for a sustained period. Despite repeatedly seeking medical help, the healthcare´s response towards disease diagnosis proved ineffective. Delays in proper treatment initiation motivated recurrent HCSB. For some interviewees, patient delay was unclear but long as they stressed different entry points explored along the healthcare pathway.

Two participants initially used PHC facilities where symptoms were underestimated given that exams were “normal”. Afterwards, one participant actively searched for private consultations; however, the diagnostic cascade was initiated at the ER, 8 months later. The other had a pre-scheduled consultation with a public hospital specialist MD who noticing extreme weight loss, triggered the diagnostic cascade for TB, 9 months after the first consultation. Another participant waiting for a referral from PHC to specialised care, turned to the ER approximately 4 months following the initial consultation at PHC.

*" I arrived in the ER, explained the case (…), because the letter at home [appointment for a consultation following referral from PHC] never arrived, really, never arrived." (P15, female, age 31, Angola)*

For one participant with multiple co-morbidities, patient delay remained undefined as this illness episode overlapped with other illnesses, masking symptoms. He waited for a pre-scheduled specialist consultation, however symptoms worsened, and he ended up in ER. Another migrant participant mentioned initial health complaints diagnosed as depression. Long after, following an abnormal chest x-ray she was advised to travel to Portugal where she underwent surgery. Some months following her last surgery, she was hospitalised; as an in-ward patient, TB was suspected.

*" I had constant complaints (…) I went to several hospitals (…) and they said everything was okay (…) Then one day…, I had a chest x-ray…, she [the MD] said I had a strange inflammation in my lungs…, gave me the number of a surgeon… and we began to hurry from that day on” (P29, female, age 25, Angola)*

Many migrant participants consulted MDs in private and public clinics, traditional medicine in their home countries (and even neighbouring countries), but no effective treatment was given. Some came to Portugal seeking better healthcare, using different entry points, i.e., a private clinic, PHC or the ER (Table 2).

Except for one participant, the common end point was the ER, followed by hospitalisation. Once more TB symptoms were unspecific, in this case associated with a prolonged deteriorating health status. The healthcare system delay ranged from four months to over one year, with missed opportunities of care. Participants were all diagnosed with extrapulmonary TB, except for one participant with pulmonary TB but initial “normal exams” at PHC level (Table 2).

In a HBM perspective, this category of HCSB suggests a high perceived severity of disease but also involves overcoming greater barriers to access effective treatment, as symptoms worsen, and the health system fails to resolve the complaint (Table 3).

### Absent HCSB—*“embraced/protected” by the system*

Six participants not actively procuring healthcare, were diagnosed with TB owing to services acting preventively. They narrated a pathway of active intervention by the system without which, illness would have remained unmasked (and untreated) for an indeterminate period. Patients attributed delay remains ill defined, as participants were asymptomatic, or did not (or minimally did) seek medical help before diagnosis. Some of them tended to express an inhibited HCSB, however available services, actively protected them from a worsening health status (Table 2).

*"I was in a refugee centre (…) I was normal, and then I went to do the x-ray and then he said I was ill” (P2, female, age 19, Angola)*

Whereas some participants remained passive throughout this process, others accessed a preventive service when symptomless. These services were occupational health, TB contact tracing or, a pre-scheduled consultation for another health problem, where “opportunistic” medical advice was given.

*"It was the doctor on the last consultation (…) I coughed near her, she looked at me:—I am not from this field (…) but if I were you, I would… do a chest x-ray” (P28, female, age 54, Brazil)*

Several health services offered protection for these participants allowing TB to be diagnosed. None of the participants had an assigned family MD. Health system delay was less than 2 months, except for P16 whose trajectory was longer as exams were done ambulatorily (Table 2). Participants suffered mainly from pulmonary/pleural TB, except for the participant who benefited from the ambulance service. For these participants, perceived susceptibility ranged from high (using a preventive service such as occupational health) to low (acting with an inhibited HSCB) and barriers to accessing healthcare were low as diagnostic pathways were clear (Table 3).

## Discussion

**Fig 2:**
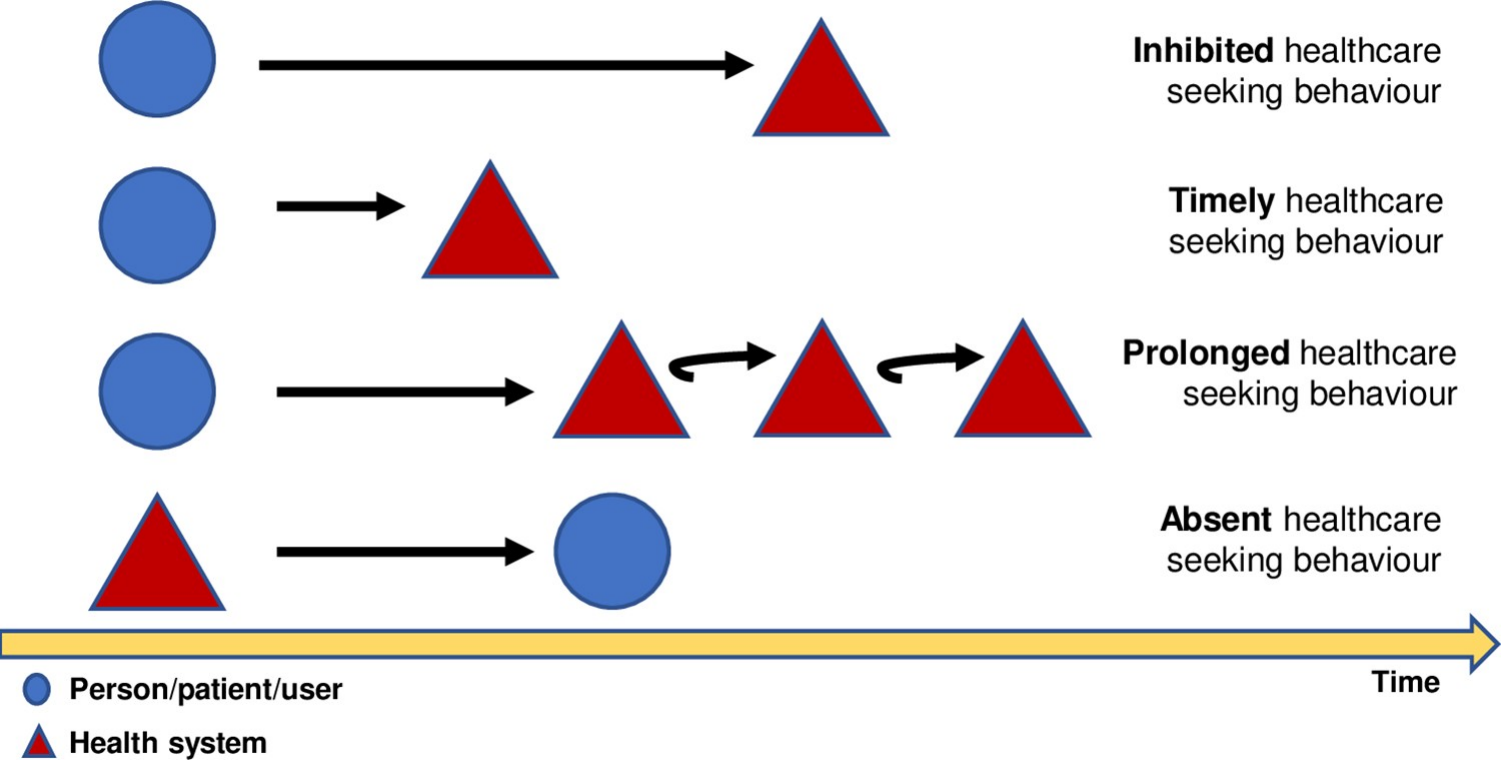
Four main types of “mindsets” of healthcare seeking behaviour for an episode of illness. We consider a dichotomic relationship between patient and the healthcare system influencing total time to treatment. Time to treatment is influenced by healthcare seeking behaviour pattern and healthcare system response.

By aiming to describe participants´ routes towards and inside healthcare for an undiagnosed illness episode (which ended up as TB), this study sheds light on the main diagnostic route (and its associated delays), for a specific setting. It also resulted in a novel conceptual model of healthcare seeking behaviour based on empirical information collected from patients. Healthcare seeking behaviour of migrants has been previously assessed in Portugal through a focus group study demonstrating patterns of regular use, emergency use and no use [40]. To advance knowledge we propose categorising HCSB into four key behavioural patterns related to how people act when faced with illness, and how the system retroactively influences this behaviour (Fig 2). This classification shows that HCSB is heterogeneous and context dependent.

This study also furthered our understanding on health care delivery in a deprived area within the Portuguese tendentially universal healthcare system. The study´s setting is a “hotspot” of TB transmission, encompassing a migrant population, with accumulated TB risk factors, among whom nationals tendentially had non-qualified jobs. In our study, recent migrants mainly originated from Angola and Guinea-Bissau, countries with high TB prevalence [1] and historical links to Portugal. Migrants and nationals were evenly present in each identified category, supporting that, patterns of HCSB tend to be similar when generated in a similar context. In our study, cultural barriers did not seem relevant in TB patients´ pathways inside the Portuguese SNS.

The “typical” TB patient accumulating risk factors, overlooking symptoms, and delaying seeking medical assistance may be classified as having an inhibited HCSB [13, 41]. In our study, participants with this mindset, delayed seeking medical help the most. Socio-economic class might play a role in increased delays for seeking medical help, which might also happen with other slow onset types of disease, such as cancer [42].

People with an inhibited HCSB seemed to “neglect”, “normalise” or “fear” disease. Similarly to the study by Craig et al. with “hard-to-reach” population, we believe structural barriers are relevant for this category, and classic TB symptoms do not trigger HCSB [13]. Their relationship with the health system is distant, healthcare service use is low, self-management of symptoms is high, and the ER is the place of choice when certain cues to action kicks in. To lessen missed opportunities from the health system for early case detection, there is a need to consider these persons’ social determinants of health and structural vulnerability [43].

The timely HCSB is associated with a high awareness and concern, resulting from disease´s initial symptoms. Participants showed an acceptable patient delay—in our study, less than a month—matching other European studies on TB delay [7]. Many of these participants were well acquainted with MDs or showed ease with the healthcare pathway. Respondents expressed their preference for entry points based upon satisfaction, familiarity, accessibility, informal knowledge, literacy, economic capacity, etc. In Portugal, one factor associated with a decreased patient delay is being HIV positive [8, 9]. Our study corroborates these data. Facilitated access to medical services associated with higher levels of illness awareness were key to a timely HCSB.

In our study, healthcare system delay for pulmonary TB was less than 1 month in most cases (including pleural TB which can be suspected by a chest X-ray), matching other studies [8]. For extrapulmonary TB (EPTB) (excepting pleural TB), delays increased drastically as EPTB itself constitutes a proved risk factor for longer healthcare system delays [8, 44].

One study shows that healthcare system delays of over 10 weeks were related to EPTB or paying the first visit to a GP [44], which we corroborate. Diagnosing EPTB is inherently difficult: the array of symptoms is varied and unspecific, basic diagnostic exams can be “normal”, and bacteriological confirmation although advisable can be challenging [45, 46]. As a result, MDs and patients tend to confuse symptoms with other ill-health conditions (especially when co-morbidities are present). Moreover, PHC entry points face difficulties in recognising atypical symptoms [44], referrals to a higher tier of the health system may linger, complex diagnostic exams may further extend waiting times, thereby all contributing to increasing delays.

In the case of migrant patients from countries with substandard health systems, cycles of unproductive healthcare and HCSB are perpetuated decreasing trust in local health services. In our study migrants travelled to Portugal in search of better treatment and diagnostic options. A prolonged HCSB converts them into “hyperusers” of healthcare. Complex health and social needs tend to remain chronically unsolved by healthcare systems, increasing the costs of care [12].

While some people may remain chronically attached to healthcare services for persistent symptoms, some services enable shortening diagnostic delays when there is an increased risk of disease. The services assisting participants in this study were: a TB screening program, an occupational health service, a specialist consultation, an ambulance service, and a charitable organisation.

Occupational health, particularly for high-risk professions (such as healthcare workers), as well as screening programs for high-risk groups (such as TB contacts or refugees), showed its importance in reducing TB delay [47]. A specialist follow-up consultation also protected participants from delayed diagnosis. One possible explanation refers to hospital specialists tending to have a higher degree of disease suspicion compared to PHC [48]. Another explanation is that a pre-scheduled consultation (instead of acting upon an urgent need), may be protective, as by contacting a healthcare professional a person is exposed to receiving “opportunistic healthcare”.

The main bottleneck of the healthcare system highlighted here is the emergency room (ER). A Brazilian qualitative study explains TB patients´ preference for the ER due to cultural factors, i.e. a fear of being recognised or stigma, the culture of immediacy and the wish for “complete” care, and failures of the basic network [49]. For our study area, the preference for the ER appears to have been affected by a difficult access to PHC. Not having an assigned MD may influence accessibility for PHC. A Canadian study, interviewing “individuals with complex health and social needs” and their caregivers, identifies the ER as an “unavoidable point-of-care” [12]. The ER is an overused source of help seeking probably attributable to its non-stop availability and its tendentially resolvent nature. Problems with PHC includes, few (if available) point-of-care exams, delays in scheduling and receiving exam results done ambulatorily, difficulties in arranging a consultation (specially for people without an assigned MD), reduced time per patient by consultation, and bureaucratic difficulties in access for migrants. All this reinforcing the tendency of both patients and health professionals to refer to the ER. However, the ER choice may increase the number of hospitalised patients.

Methodologically, the HBM was used here as a reference framework to consolidate the proposed model of HCSB. It allowed to cross-pollinate ideas between the patient´s relationship with the healthcare system (inhibited, timely, prolonged, or absent), and their individual perceptions of illness, adding value to the proposed new model of HCSB. Participants´ narrative served as the conductive line to dialogue between conceptual components of the HBM and the empirical findings.

The main limitation of this study and its main strength is its qualitative nature; it relies on people´s narratives submitted to a recall bias. Aiming to reduce it, we selected patients on active treatment. However, by interviewing the successfully treated patients with sensitive TB attending pre-scheduled consultations, we excluded default patients, resistant TB patients and undiagnosed ones. Furthermore, the fact that TB might touch upon sensitive issues led some patients to refuse to participate or audio-record their statements. The fact that the interviewer was an MD facilitated cooperation for some participants.

In the analysis stage, the compatibility of participants’ behavioural patterns within each category was carefully assessed, as life stories are not rigid happenings. Regarding the use of the HBM, the construct of self-efficacy was not explored here. Although its role in shaping HCSB is likely relevant, participants’ pathway narratives did not disclose enough information to “theorise” on this construct. Based upon our qualitative data, no correlation between socio-economic status and HCSB categories could be established, which would justify further study.

Despite its limitations, the study´s qualitative nature allowed for encountering explanations for people´s behaviour based on their own narratives, contrary to quantitative approaches. It thus enabled an understanding of their circuits and the detection of key points where quality improvements are needed in order to reduce diagnostic delays. We were also able to show how non-medical issues mediates the experience of healthcare services.

By using empirical research to underpin theoretically informative concepts, qualitative data enabled developing a novel approach which better contextualises interactions between patients’ and services. By building a theoretically grounded HCSB model we encourage further research to test its generalizability in other settings.

## Conclusions

TB is a complex disease strongly associated with social determinants of health. By analysing patients´ circuits, we identified four types of HCSB associated with delays in treatment initiation. Nationals and migrants showed similar behavioural patterns suggesting life cycles and contexts matter more than places of origin. Possible structural barriers associated to an inhibited HCSB should be addressed, as well as complex medical needs of people expressing a prolonged HCSB. People with a timely disease diagnosis represents an optimal diagnostic pathway that should be promoted. Boosting preventive services helps reducing community´s infectiousness by lowering the threshold of disease detection. Improving quality of primary healthcare service may contribute to addressing complex health and social needs and reduce the burden of patients in the emergency room.

## Supporting information

S1 Appendix Interview topic guide. (DOCX)

S2 Appendix COREQ checklist. (PDF)

S3 Appendix Exhaustive compilation of quotes supporting healthcare seeking behaviour types. (DOCX)

S1 File Abstract in Spanish. (DOCX)

S2 File Abstract in Portuguese. (DOCX)
