# Supplementary material for: The circuits of healthcare: Understanding healthcare seeking behaviour—A qualitative study with tuberculosis patients in Lisbon, Portugal
Source: PLoS One. 2021 Dec 28;16(12):e0261688. doi: 10.1371/journal.pone.0261688 (PMC8714083; doi:10.1371/journal.pone.0261688)
Supplement: S1 Appendix — (DOCX) [file pone.0261688.s001.docx]

**Topic guide for semi-structured interview**

**with TB patients**

**General behavior when sick**

1. Imagine a situation where you got sick (or a family member, your children), how was it? What did you do?
2. Regarding the previous situation, where did you search for help? How did you go to the place (transport)? Which exams did you do and how was it (cost, waiting time, complexity)? How did you get the medicines?
3. How would you qualify the healthcare you received? (waiting times, satisfaction…)

**TB Illness onset**

1. Tell me how was the beginning of this illness episode? How was it for you?
2. Where did you search for help? (consultations, exams, treatment, family support)
3. What were the main difficulties you have faced? (transport, financial, literacy…)
4. What has helped you in this process?

**Times**

1. Since you started feeling sick until the doctors told you were sick of tuberculosis how long has it passed? Tell me how did you live this period of your life, how was it for you?

**Reaction when faced with TB diagnosis**

1. What did you feel when doctors told you this diagnosis?
2. Who was the first person you told the diagnosis? How was it?
3. How was it at work?
4. What have been the most difficult for you in this process?
5. What has helped you the most?

**Satisfaction with the healthcare service**

1. What do you think about the healthcare you have received? (3 positive and 3 negative aspects)
2. Is there something you would like to be different?

**Specific questions for the purposive sample of migrants**

**The decision to migrate**

1. What are the motifs leading you to migrate, can you explain it to me?
2. Which reasons influences the choice of Portugal?
3. How was the preparation process for the trip (work, visa…)? How long did it take?
4. What were the main obstacles that emerged in the process?
5. Which help/ support did you get? (work, family, institutions…)

**Arrival to Portugal**

1. How was the arrival in Portugal?
2. When did you have the first consultation in Portugal? And where was it?
3. Did you have to go to the migration office? Tell me how it was.
4. In this period, you have been in Portugal, which were the main difficulties you faced? Tell me 2 or 3.
5. What has helped you the most?
6. What do you miss the most in your country? Are you organizing the return home?
