# Supplementary material for: The circuits of healthcare: Understanding healthcare seeking behaviour—A qualitative study with tuberculosis patients in Lisbon, Portugal
Source: PLoS One. 2021 Dec 28;16(12):e0261688. doi: 10.1371/journal.pone.0261688 (PMC8714083; doi:10.1371/journal.pone.0261688)
Supplement: S3 Appendix — (DOCX) [file pone.0261688.s003.docx]

| **Reasons explaining each healthcare seeking behaviour type** | **Quotes** | **Participants: gender, age and country of origin** |
| --- | --- | --- |
| **Inhibited HCSB** |  |  |
| Fear of hospitals and of the possible implications of diagnosis | *" I knew I was sick but I didn´t want to go to the hospital… maybe I was afraid… of knowing… I started to lose weight suddenly.”* | *P8, male, age 28, Portugal* |
| The patient surrendering to the situation created by illness and assuming an attitude of inertia | *“I just stayed at home sleeping, nothing more”.*  *"I had been thinking for 2-3 months that it was a pulmonary disease, a flu… that it would go away… until the point I could not go up the stairs”.* | *P9, male, age 22, Guinea-Bissau*  *P5, male, age 42, Portugal* |
| The need to work to cover financial expenses and alcohol masking symptoms | *" Life is not that easy for us to be missing working days… by that time my wife was unemployed, and with 2 kids… I have bills to pay, I never went to the hospital… by that time, I also drank, I drank a lot, maybe the alcohol itself masked the deficiency the body had… because of the alcohol a person was not aware of the body.”* | *P26, male, age 45, Portugal* |
| Stigma associated with HIV infection | *" I was afraid… I always have this fear of rejection, you know? When he told me: - Show me the report from Brazil, I was frightened, I said - I haven´t brought it, I am going to bring it next time. Then I had to bring it along today, but with lots of fear of how I was going to be received.” [This participant was diagnosed with HIV then with TB in Brazil and came to Portugal interrupting treatment.]* | *P3, female, age 36, Guinea-Bissau* |
| Cues to action |  |  |
| Extreme pain and fear associated with it | *“… the cough was becoming compulsive, but it was mainly because of the chest pain and it took the arm also, then I was a bit scared”.* | *P26, male, age 45, Portugal* |
| Societal “push” | *“… there was a woman on the coffee place I usually go, she said “You go to the doctor because this is not normal… you are eating a third of what you used to eat… whether you go to the hospital, whether I will go there with you”. On the other day I took the car and went to the hospital”.* | *P5, male, age52, Portugal* |
| Societal “push” | *“I thought it was only a flu, I ignored everything, I had a cough, 2 months, I was already feeling ashamed in the bus.”* | *P3, female, age 36, Guinea-Bissau* |
| **Timely HCSB** |  |  |
| Accessibility to a doctor - specialist consultation | *" I have been around a week feeling this way then I went to the hospital… normally I go to the hospital where I received follow-up treatment for HIV because they have my clinical history and normally when I feel weak it is because of the illness.”* | *P11, male, age 39, Portugal)* |
| Accessibility to a doctor -  doctor who is a friend working in a hospital | *"We called a doctor we know who works in the hospital of Santa Maria, and she said: “it is better for her to come”* | *P22, female, age 20, Portugal* |
| Accessibility to a doctor -  an assigned family physician well-known to the participant | *"I went to the healthcare centre, the doctor that assists me wrote a document to come to this clinic. I am with this doctor for about 15-20 years”* | *P13, male, age 81, Portugal* |
| Accessibility to a doctor -  emergency hospital consultation | *"… because my university is associated with the hospital of Santa Maria… it was what I believed to be the ideal place to be assisted, because of proximity.”*  *"It was three in the morning when I woke up with an intense pain, I went directly to the hospital* | *P7, male, age 32, Angola*  *P6, male, age 52, Portugal* |
| Accessibility to a doctor -  Private health service | *" I started to have a problem in the leg, I began to limp, I felt pain, then I searched for a physiotherapist and did physiotherapy… after my feet began to swell (…) I had an appointment with physiotherapist, and he advised me to go to the emergency room.”*  *"I arrived [in Portugal] and I went directly to a consultation [in a private hospital], I already knew it. I had done my consultations there, my surgery was done there too, my c-section and the vocal cords nodules. I have always done my consultations there”* | *P27, male, age 25, Angola*  *P23, female, age 33, Angola* |
| **Prolonged HCSB** |  |  |
| Primary healthcare underestimated symptoms given that exams were “normal” | *“What I felt was an unease here on the side, I went to the healthcare centre because of that, I had many consultations (…) I did exams, consultations, finally I was referred to the specialist at the hospital… then, months had passed, I went to an osteopath… but after some months I began feeling headache again, then I went directly to the ER”.*  *" Before doing the exam of TB, because I have been suffering, the doctor [family MD] did not ask me to do anything [more exams], the analysis was all fine, the exams were all good, there was nothing there to be seen”.* | *P25, male, age 33, Portugal*  *P10, female, age 86, Portugal* |
| Multiple co-morbidities masking symptoms and “normal exams”  Waiting for a pre-scheduled specialist consultation and exams | *"Mainly fatigue. A lot of fatigue. A hold on the chest, but I have a heart problem, it has been detected, as a patient I can´t know (…) It has been some years, more than five, I began to lose weight… even in bed I felt without energy… but after, exams looked okay (…) I was working and I didn´t want to stop working (…) I had a previously scheduled consultation, I had a CT-scan for the gut but, as I went to emergency room, procedures were followed-up in another hospital”.* | *P14, male, age 56, Cape Vert* |
| Waiting for a referral letter from primary healthcare | *"I went to the ER, I arrived in the ER, explained the case, they gave me a paper for me to go to the day hospital of infectiology. I went there, they themselves scheduled the consultation, from that moment on I have been following consultations until now, because the letter at home never arrived, really, never arrived. It was through the ER that I got it."* | *P15, female, age 31, Angola* |
| Health services of countries of origin were unable to solve the illness situation | *"… and I remained with that back pain, back pain, I could not get up from the bed, could not walk, I went to [another country] to do treatment, one month of treatment in [another city] … I began to feel better, went back to Guinea [Bissau], less than two weeks passed, and pain started again, the same severe pain, then my husband said I could not continue with that pain and I paid to start treatment here in Portugal”.*  *"I could not do the things I used to do, in my daily life, like cooking, organising… I could not walk any distance (…) I went to traditional medicine… consultations were expensive, but I… sometimes I did not mind spending money, the importance was to be fine. Even then, nothing! (…) I was coming from another country, to here, for treatment, there are documents to be arranged to start consultations. We went to emergency room and I explained my case”.* | *P4, female, age 35, Guinea-Bissau*  *P20, female, age 32, Angola* |
| Possibility of a missed/wrong diagnose | *" I had constant complaints, I complained about tiredness, lack of appetite, afterwards came weight loss, and I had lots of dry cough. I went to several hospitals to know what was it, also the hospital in XXX [another country], and they said everything was okay, because I just lost my father (…) Then one day (…) then I went to a clinic to check that, I had a chest x-ray… she said I had a strange inflammation in my lungs that she could not say more but it was very important. She said I had to have surgery, gave me the number of a surgeon and we began to hurry from that day on”.* | *P29, female, age 25, Angola* |
| Exams done ambulatorily instead of in-ward condition (hospitalisation) | *"I said – Well doctor, ask me anything, but don´t ask me to be hospitalised, I will do anything you ask me, I can come every day here if necessary, the time that is needed… -So then, okay! Come here on this day to do an exam (…) I arrived there, I didn´t even know, everybody was on strike!".* | *P10, female, age 86, Portugal* |
| **Absent HCSB** |  |  |
| Initial inhibited HCSB that will be embraced by the health system | *" I think they sent me to the Healthcare centre, as I said I was feeling sick. They said – go to the healthcare centre, but to go the healthcare centre I had to wake up very early and wait in a queue. Then I said, it´s okay, it will pass.”*  *"I was thinking it was a flu I had, so I left it until the point I was taking medication lots of times, I did not go to the healthcare centre, or the hospital, neither anything, to be cured. I was certain thinking it was flu, I went to the pharmacy and bought medication to take”.*  *“I knew the symptoms of tuberculosis, but the symptoms I had did not identify anything. I identified it as a strong flu, it was a constant cough. Not everyone who coughs has tuberculosis, because then all humanity would be condemned, right?” .* | *P2, age 19, Angola*  *P17, female, age 34, Portugal*  *P28, female, age 54, Brazil* |
| Screening program | *"I was in a refugee centre, and there, they made some exams in people to know if everything is okay. I had an x-ray and then the doctor saw there was something wrong… I was normal, I was like this, and then I went to do the x-ray and then he said I was ill”.* | *P2, female, age 19, Angola* |
| Ambulance service | *“I fell in the shop of my brother-in-law. We were there, having lunch, sitting, chatting, everything was okay. I fell. The ambulance came and took me to the hospital”.* | *P19, male, age 33, Guinea-Bissau* |
| Occupational health | *“It was a routine exam, a routine x-ray at the occupational health… the doctor there said there was something she did not know… I said – Look I do x-rays every year, and I didn´t have anything… Because I am a smoker, so I am a little bit concerned (…) She made a referral".* | *P16, female, age 33, Portugal* |
| Pre-scheduled follow-up specialist consultation | *"It was the doctor on the last [specialist] consultation I had... I coughed near her, and when I asked to cough and put away the spittle because I coughed so much that I choked, she looked at me: - I am not from this field, I am not a pneumologist, but if I were you, I would come back here today or tomorrow and do a chest x-ray…”.* | *P28, female, age 54, Brazil* |
| “Push” to seek healthcare done by a charity organisation lodging one participant | *"… because me alone, if I was alone, if I was at my house, I would go to the pharmacy… and buy, I would not go to the hospital or anything, but as I was in the place where I am, I had to go to the health care centre or hospital, to know what was happening”.* | *P17, female, age 34, Portugal* |
