# Supplementary material for: The circuits of healthcare: Understanding healthcare seeking behaviour—A qualitative study with tuberculosis patients in Lisbon, Portugal
Source: PLoS One. 2021 Dec 28;16(12):e0261688. doi: 10.1371/journal.pone.0261688 (PMC8714083; doi:10.1371/journal.pone.0261688)
Supplement: S1 File — (DOCX) [file pone.0261688.s004.docx]

**“Los circuitos de los servicios de salud: comprendiendo los comportamientos de búsqueda de cuidados de salud - un estudio cualitativo con enfermos de tuberculosis en Lisboa, Portugal”**

**Resumen**

**Contexto:** Comprender los servicios de salud desde una perspectiva del paciente, incluyendo factores que influencian los comportamientos de búsqueda de cuidados, es crucial para tratar enfermedades, sobre todo infecciosas, como la tuberculosis. Este estudio pretende rastrear y contextualizar las trayectorias de las personas hacia el diagnóstico y tratamiento, y discutir sobre factores clave asociados al retraso del inicio del tratamiento. Las trayectorias de los enfermos de tuberculosis, sirve como indicador de las dificultades de los más vulnerables en la obtención de cuidados de salud adecuados.

**Métodos:** Hemos realizado 27 entrevistas semiestructuradas con enfermos de tuberculosis en un centro de tratamiento en Lisboa. Hemos invitado pacientes nacionales y migrantes en tratamiento activo han a participar, compartiendo sus experiencias desde el inicio de los síntomas hasta el momento de la entrevista. El Modelo de Creencias de Salud fue utilizado como referencia teórica para consolidar los hallazgos cualitativos.

**Resultados:** A través del análisis inductivo de las entrevistas, hemos categorizados el comportamiento de búsqueda de cuidados de salud en 4 tipos (inhibido, oportuno, prolongado y ausente). Cada tipo de comportamiento expresa una actitud que influencia la forma que los participantes procuraron los servicios de salud. La principal puerta de entrada fue el Servicio de Urgencias. La Atención Primaria fue infrautilizada.

**Conclusiones:**  Los resultados respaldan que el comportamiento de búsqueda de cuidados de salud no es homogéneo e influencia los retrasos diagnósticos. Si deseamos reducirlos, debemos considerar diferentes patrones de comportamiento en el diseño de medidas para mejorar los servicios de salud. Los profesionales de salud deben sensibilizarse y recibir formación continua para tratar a los enfermos. El comportamiento inhibido o prolongado contribuye significativamente a los retrasos diagnósticos. Estos deben ser detectados y revertidos. Respuestas oportunas del sistema y del enfermo, deben ser promovidas.
