# Supplementary material for: The circuits of healthcare: Understanding healthcare seeking behaviour—A qualitative study with tuberculosis patients in Lisbon, Portugal
Source: PLoS One. 2021 Dec 28;16(12):e0261688. doi: 10.1371/journal.pone.0261688 (PMC8714083; doi:10.1371/journal.pone.0261688)
Supplement: S2 File — (DOCX) [file pone.0261688.s005.docx]

**“*Os circuitos dos cuidados*: entendendo os comportamentos de procura de cuidados de saúde – um estudo qualitativo com pacientes doentes de tuberculose em Lisboa, Portugal”**

**Resumo**

**Contexto:** Entender a prestação de cuidados de saúde desde uma perspetiva dos doentes, incluindo fatores que influenciam o comportamento de procura de cuidados, é crucial no tratamento de doenças, sobretudo infeciosas como a tuberculose. Este estudo pretende identificar e contextualizar as trajetórias percorridas pelos doentes rumo ao diagnóstico e tratamento, abordando fatores chave relacionados com o atraso no início do tratamento. Os circuitos dos doentes de tuberculose, serve como indicador das dificuldades dos mais desfavorecidos na obtenção de cuidados de saúde adequados.

**Métodos:** Realizamos 27 entrevistas semiestruturadas com paciente de tuberculose num centro de tratamento na zona periférica de Lisboa. Convidamos pacientes nacionais e migrantes em tratamento ativo para participar partilhando suas experiências desde o início da doença até o momento da entrevista. O Modelo das Crenças de Saúde foi utilizado como referência teórica na consolidação dos resultados qualitativos.

**Resultados:** Por análise indutiva das entrevistas, categorizamos o comportamento de procura de cuidados de saúde em 4 tipos (inibido, atempado, prolongado e ausente). Cada tipo expressa uma atitude que influencia a forma que os participantes atuaram perante a doença. O Serviço de Urgência foi a principal porta de entrada para o início da cascada diagnóstica, e os Cuidados de Saúde Primários foram subutilizados.

**Conclusões:** Os resultados apoiam que o comportamento de procura de cuidados de saúde não é homogéneo, e influencia os atrasos diagnósticos. Se desejamos diminuir os atrasos, a identificação de padrões de comportamento deve ser considerada no desenho de medidas para melhorar a prestação de cuidados de saúde. Profissionais de saúde devem estar sensibilizados e receber formação contínua para lidar com as necessidades dos doentes. Os comportamentos tipo inibido ou prolongado contribuem significativamente para os atrasos diagnósticos. Estes devem ser detetados e revertidos. As respostas atempadas, por parte dos doentes e do sistema de saúde, devem ser promovidas.
